# Severe malaria in Canada, 2001–2013

**DOI:** 10.1186/s12936-015-0638-y

**Published:** 2015-04-11

**Authors:** Anne E McCarthy, Chardé Morgan, Chatura Prematunge, Jennifer Geduld

**Affiliations:** Division of Infectious Diseases, The Ottawa Hospital, 501 Smyth Road, Ottawa, ON K1H 8L6 Canada; Public Health Ontario, Ottawa, Canada; Travel and Migration Health Division, Public Health Agency of Canada, Ottawa, Canada

**Keywords:** Severe malaria, Imported malaria, Artesunate, Quinine

## Abstract

**Background:**

Imported malaria is the principal, preventable, life-threatening infection among Canadians travelling abroad. The Canadian Malaria Network supplies information and parenteral malaria therapy to healthcare providers treating severe and complicated malaria and gathers surveillance information on these cases.

**Methods:**

Data were collected on the characteristics, risk factors, and clinical outcomes of severe malaria cases in Canada from June 2001 to December 2013.

**Results:**

The need for parenteral therapy in Canada has increased in the last decade. The vast majority of cases are reported from Ontario and Quebec and occur among travellers to and from Africa. Regardless of country of birth, all persons originating from endemic and non-endemic countries are at a similar risk of malaria-related complications. Overall use and appropriateness of pre-travel advice and chemoprophylaxis remains low. Most cases result from patient delays in recognizing symptoms and seeking appropriate medical attention. Although some healthcare delays occurred in a select number of cases, the majority of patients were diagnosed quickly and were appropriately treated with parenteral therapy within a few hours of diagnosis.

**Conclusions:**

Data from the Canadian Malaria Network provide insight into the characteristics of imported severe and complicated malaria infections in Canada. Improved understanding of this population can help target risk reduction strategies and interventions to limit personal susceptibility and healthcare treatment delays.

**Electronic supplementary material:**

The online version of this article (doi:10.1186/s12936-015-0638-y) contains supplementary material, which is available to authorized users.

## Background

Malaria is the principal, life-threatening, infection among international travellers from Canada, and a frequent health concern for individuals travelling between Canada and endemic regions. The majority of malaria infections in Canada are imported and occur in new immigrants or Canadians travelling to their region of origin to visit friends and relatives (VFR) [1,2]. In 2009, 35% of Canadian travellers bound to destinations other than the USA visited regions with a risk of malaria, a 131% increase from 2000 [3]. Between 1991 and 2011, an average of 483 (range: 333–1032) malaria cases per year were reported to the Public Health Agency of Canada [4]. Many of these infections can be prevented through pre-travel healthcare consultation, appropriate use of chemoprophylaxis, and personal protective measures to prevent mosquito bites. Moreover, the consequences of infection can be minimized through health care provider knowledge of disease symptoms, early diagnosis and treatment.

Risk of severe malaria is associated with *Plasmodium falciparum* infection which progresses to severe disease, leading to clinical complications such as coma, renal failure, respiratory distress, or death [5]. Severe malaria infection can occur when malaria is not treated rapidly with appropriate anti-malarial therapy. Parenteral artesunate, introduced to Canada in 2009, is the preferred medication for the treatment of severe malaria due to *P. falciparum*. Parenteral quinine is the alternative therapy when artesunate is contra-indicated, or when the only indication for parenteral therapy is failure to tolerate oral therapy. Both drugs are made available in Canada via the Canadian Malaria Network (CMN) through Health Canada’s Special Access Programme that allows practitioners to request access to drugs that are unavailable for sale in Canada [6-8].

The CMN is a national network of designated medical centres of malaria expertise across Canada that supply information, rapidly distribute parenteral malaria therapy and collect surveillance data on severe and complicated malaria [6]. No research to date has collectively examined malaria infections requiring parenteral therapy in Canada. Therefore, with the aim of better understanding imported severe malaria infections in the region, surveillance data from CMN between 2001 and 2013 were analyzed to describe national trends, risk factors and delays associated with accessing parenteral anti-malarial therapy across Canada.

## Methods

### Data collection

Data for cases accessing parenteral therapy were reported to CMN’s coordinating site. Attending physicians are required to complete two case report forms, which are supplied with the treatment medication [see Additional file 1] Form A collects non-nominal information on patient demographics, travel history, infection, and treatment delays on day 1 of treatment. Form B collects follow-up information on disease outcome (s), health care requirements, drug utilization and adverse events seven days following initial treatment administration. Both forms are available on the CMN website.

### Definitions

Severe malaria is defined by the clinical features established at the World Health Organization (WHO) that includes: History of recent possible exposure and no other related pathology OR asexual forms of *Plasmodium falciparum* on blood smear AND any one or more of the following features: hyperparasitemia (>2% in non-immune, >5% in semi-immune); impaired consciousness or coma; prostration (unable to walk or sit up without assistance); multiple convulsions (>2 in 24hrs); respiratory distress (acidotic breathing); respiratory failure/pulmonary edema/ARDS; circulatory collapse/shock (SBP<80mmHg adults and <50mmHg children); acute kidney injury/renal failure (Cr >265μmol/L or >upper limit for age for children); jaundice (total bilirubin >45μmol/L); abnormal spontaneous bleeding/DIC; hypoglycemia (<2.2mmol/L); metabolic acidosis/acidemia (pH<7.25, HCO3<15mmol/L); severe anemia (< 50g/L, in children; <70g/dL, in adults); hemoglobinuria (macroscopic); hyperlactataemia (lactate >5mmol/L) [7]. Complicated malaria is defined as malaria infection in an individual who cannot tolerate oral treatment (e.g., vomiting), without clinical features of severe disease.

All case reports submitted to CMN were reviewed by a tropical medicine physician (AM) to establish treatment and chemoprophylaxis appropriateness [7]. Appropriate indication for parenteral anti-malarial treatment was classified as complicated malaria or severe malaria infection [7]. Chemoprophylaxis regimen appropriateness was established by travel destination and prevention guidelines published by the Committee to Advise on Tropical Medicine and Travel (CATMAT) [9].

Patient birth countries were classified as malaria-endemic (mBC) or non-malaria-endemic (non-mBC) using the WHO list of malaria-endemic countries [10].

Patient delays were defined as days between symptom onset and initial Canadian physician visit. Healthcare system delays were defined as: 1) days between the first physician visit and laboratory-confirmed malaria diagnosis; 2) hours between laboratory-confirmed diagnosis and CMN treatment request; 3) hours between treatment request and treatment receipt; and, 4) hours between treatment receipt and treatment administration. All delays are described as median values.

Therapy-related adverse events were classified as minor, serious or other. Minor adverse events were defined as symptoms of fatigue, dizziness and occasional tinnitus. Serious adverse events were defined as haemolysis, hypotension and delayed hemolysis. Delayed hemolysis was defined as hemolysis that occurred 1 - 3 weeks after initiation of treatment. Other adverse events were considered to be adverse outcomes unrelated to parental anti-malarial therapy alone and classified by the tropical medicine physician (AM) reviewing case forms.

### Data analysis

Data analysis was performed using Stata 13 [11]. Cases were separated by patient birth country and IV anti-malarial drug type. Results were compared using *t*-test, Chi-square test and Fisher’s exact test. Statistical significance was set at *p* <0.05.

## Results

Between June 2001 and December 2013, 293 patients accessed IV quinine and/or artesunate treatment through CMN. Of these, 248 (85%) were treated for severe malaria, 31 (11%) for complicated malaria and 14 (5%) had neither severe nor complicated malaria and were classified as inappropriately treated. Overall, 95% (279) of parenteral treatment requests to CMN were appropriate (Table 1). Unless otherwise indicated, the remaining results focus on patients with severe malaria.

**Table 1:** **Malaria cases treated through the CMN by appropriateness of IV therapy**

**Year**	**Total cases no.**	**IV therapy appropriate**				**IV therapy inappropriate no. (%)**	
		**Severe malaria no. (%)**		**Complicated malaria no. (%)**			
2001	15	13	(87)	2	(13)	0	(0)
2002	7	7	(100)	0	(0)	0	(0)
2003	13	8	(62)	3	(23)	2	(15)
2004	20	14	(70)	5	(25)	1	(5)
2005	12	11	(92)	0	(0)	1	(8)
2006	16	13	(81)	3	(19)	0	(0)
2007	16	13	(81)	2	(13)	1	(6)
2008	20	16	(80)	4	(20)	0	(0)
2009	23	20	(87)	3	(13)	0	(0)
2010	34	30	(88)	2	(6)	2	(6)
2011	30	25	(83)	5	(17)	0	(0)
2012	33	29	(88)	0	(0)	4	(12)
2013	54	49	(91)	2	(4)	3	(6)
TOTAL	293	248	(85)	31	(11)	14	(5)

### Severe malaria

**Figure 1:**
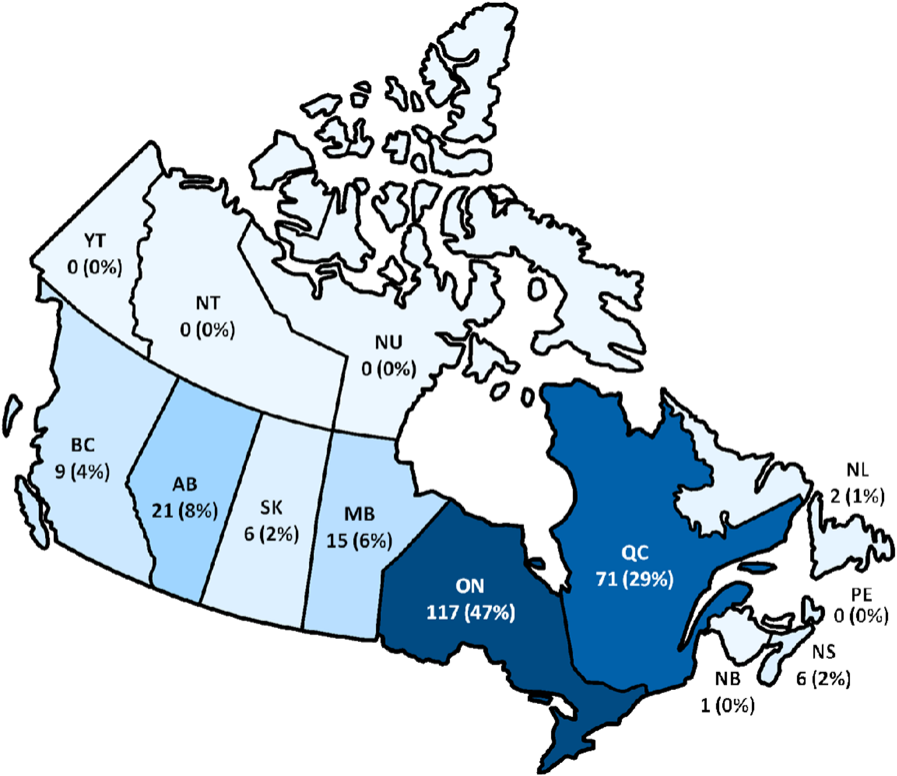
Distribution of severe malaria cases by Canadian province and territory (n = 248). Legend: AB= Alberta, BC = British Columbia, MB = Manitoba, NB = New Brunswick, NL = Newfoundland & Labrador, NS = Nova Scotia, NT = Northwest Territories, NU = Nunavut, ON = Ontario, PE = Prince Edward Island, QC = Quebec, SK = Saskatchewan, YT = Yukon.

The overall incidence of severe malaria in Canada during the study period was 54 cases per 1,000 surveillance days. There was a gradual increase in annual incidence over time with the greatest number of cases occurring in 2013 (Table 1). Most Canadian provinces, with the exception of Prince Edward Island and the Territories, have requested treatment for one or more cases of severe malaria. The majority of requests (76%) occurred in Ontario and Quebec (Figure 1). Most requests for IV therapy (82%) occurred in cities with a CMN site or satellite site.

### Demographics and risk factors

**Figure 2:**
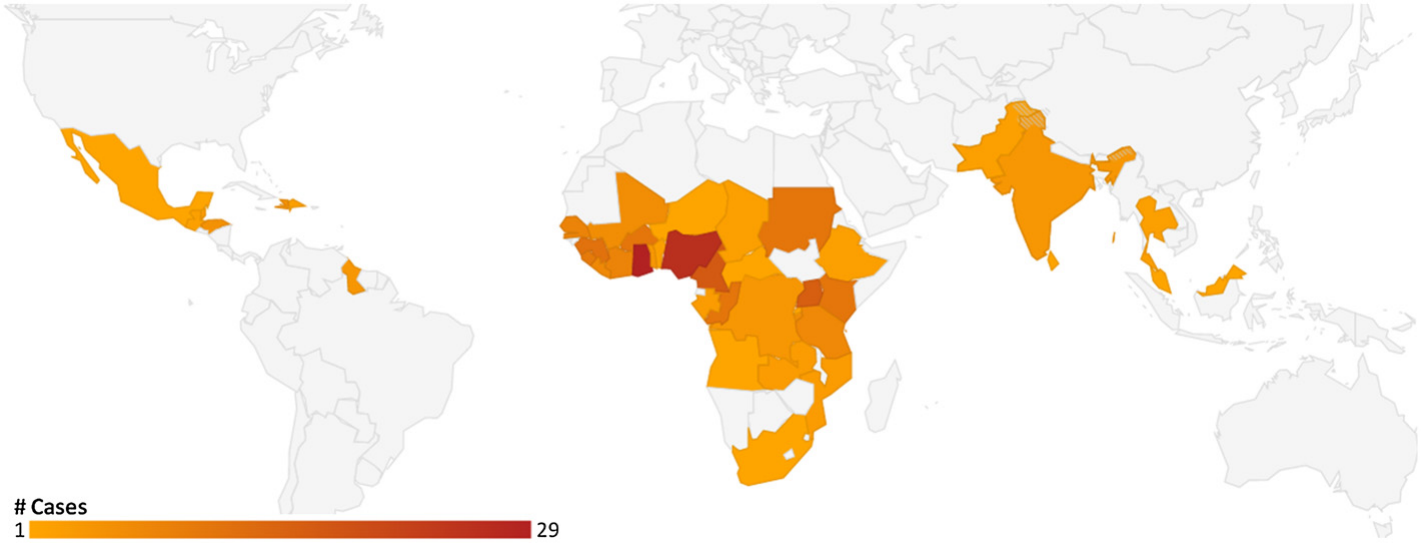
Severe malaria cases in Canada, by country of acquisition (n = 246).

Severe malaria case demographic characteristics and travel-related risk factors are summarized in Tables 2 and 3. The majority of severe malaria cases (53%) occurred among mBC by visits to and from Africa (89%). The greatest percentage of cases originated from Ghana (12%), Nigeria (11%), Cameroon (7%) and Uganda (7%) (Figure 2). *Plasmodium falciparum* accounted for 94% of severe malaria infections. Although 36% accessed pre-travel advice and 23% reported chemoprophylaxis use, only 55% of chemoprophylaxis regimens were classified as appropriate.

**Table 2:** **Socio-demographics of severe malaria cases**

	**All severe cases no./no. (%)**		**non-mBC no./no. (%)**		**mBC no./no. (%)**		**P value**
Male	137/245	(56)	70/115	(61)	67/130	(52)	0.142
Age, years, median (range)	38	(0–77)	41	(0–77)	33	(1–74)	0.002
Children (<18 years)	45/248	(18)	15/116	(13)	30/132	(23)	0.046
Residency							
Canadian resident	197/245	(80)	109/115	(95)	88/130	(68)	0.000
Recent immigrant^a^	29/245	(12)	0/115	(0)	29/130	(22)	0.000
Visitor	16/245	(7)	3/115	(3)	13/130	(10)	0.019
Other	3/245	(1)	3/115	(3)	0/130	(0)	0.064

*Abbreviations*: *non-mBC* not born in malaria-endemic country, *mBC* born in malaria-endemic country.

^a^A recent immigrant was defined as an individual who immigrated within the last year.

**Table 3:** **Travel characteristics among severe malaria cases**

**Characteristic**	**All severe cases no./no. (%)**		**non-mBC no./no. (%)**		**mBC no./no. (%)**		**P value**
Reason for travel							
Visiting friends and relatives	97/214	(45)	16/89	(18)	81/125	(65)	0.000
Business	40/214	(19)	34/89	(38)	6/125	(5)	0.000
Immigration	34/214	(16)	1/89	(1)	33/125	(26)	0.000
Vacation	29/214	(14)	23/89	(26)	6/125	(5)	0.000
Volunteer	20/214	(9)	17/89	(19)	3/125	(2)	0.000
Education	9/214	(4)	3/89	(3)	6/125	(5)	0.608
Military	0/214	(0)	0/89	(0)	0/125	(0)	-
Medical	0/214	(0)	0/89	(0)	0/125	(0)	-
Other	8/214	(4)	4/89	(4)	4/125	(3)	0.623
Region of travel							
Africa	220/248	(89)	100/116	(86)	120/132	(91)	0.243
Central & South America	10/248	(4)	7/116	(6)	3/132	(2)	0.133
Caribbean	7/248	(3)	3/116	(3)	4/132	(3)	0.833
Asia	9/248	(4)	4/116	(3)	5/132	(4)	0.887
Oceania	2/248	(1)	2/116	(2)	0/132	(0)	0.130
Length of travel, days, median (range)	31	(4–946)	31	(4–536)	35	(5–946)	0.599
Pre-travel advice obtained	78/216	(36)	55/98	(56)	23/118	(19)	0.000
Chemoprophylaxis used	55/239	(23)	35/112	(31)	20/127	(16)	0.000
Chemoprophylaxis appropriate	30/55	(55)	20/35	(57)	10/20	(50)	0.791
Infection species							
*P. falciparum*	229/244	(94)	105/114	(92)	124/130	(95)	0.287
*P. vivax*	9/244	(4)	5/114	(4)	4/130	(3)	0.588
*P. malariae*	1/244	(0)	1/114	(1)	0/130	(0)	0.285
*P. ovale*	2/244	(1)	1/114	(1)	1/130	(1)	0.926
*P. knowlesi*	0/244	(0)	0/114	(0)	0/130	(0)	-
Unknown	10/244	(4)	6/114	(5)	4/130	(3)	0.390

*Abbreviations*: *non-mBC* not born in malaria-endemic country, *mBC* born in malaria-endemic country.

There were no statistically significant differences among severe malaria cases of non-mBC and mBC with respect to region of exposure, appropriateness of chemoprophylaxis regimens, species of infection or time to parasite clearance. However, mBC cases were younger (median age mBC 33 years *vs* non-mBC 41 years), less likely to have accessed pre-travel advice (mBC 19% *vs* non-mBC 56%), less likely to use chemoprophylaxis (mBC 16% *vs* non-mBC 31%) and more likely to report VFR (mBC 65% *vs* non-mBC 18%) as the predominant reason for travel. Although not statistically significant, a greater proportion of mBC travellers were children, female and reported a longer median length of travel.

### Diagnosis and treatment delays

Patient and healthcare system delays are summarized in Table 4. The median time between landing in Canada to illness was seven days. Patients waited a median of three days following symptom onset to seek medical treatment.

**Table 4:** **Time delays associated with access to health care and treatment among severe malaria cases**

**Delay**	**Mean**	**Median**	**SD**	**Range**
Patient (days)				
Date of illness to first MD visit (n=229)	4.2	3.0	5.3	(0–50)^a^
Healthcare system (hours)				
Time from MD visit to lab diagnosis (n=229)	30.2	0.0	94.94	(0–864)^b^
Time from diagnosis to contact CMN (n=90)	2.0	0.8	4.6	(0–24)
Time from contact to receive IV drug (n=133)	2.2	1.5	2.8	(0–24)
Time from receipt to drug administration (n=18)	1.2	1.0	1.1	(0–3.5)
*Total healthcare system delay (n=248)*	*29.9*	*2.0*	*91.6*	*(0–864)* ^*c*^

*Abbreviations*: *SD* standard deviation.

^a^Two cases had delays of greater than 30 days.

^b^Five cases had delays of 9 days to a maximum of 35 days. Delays due to: P.vivax infection, unavailability of microbiologists.

^c^Elevated numbers for the same reason as explained in^b^.

Following first presentation to a Canadian physician, most infections were laboratory confirmed within 24 hours. A 45-minute delay occurred between diagnosis and parenteral treatment request from CMN, a 1.5-hour delay occurred between treatment request from CMN and receipt of IV drug and a 1-hour delay occurred from receipt of the drug to treatment administration. Collectively, these delays result in a median healthcare system delay of two or more hours from presentation to treatment.

A clinically significant difference in median time from treatment request and receipt of IV drug was observed between cases presenting in cities with a CMN site or satellite centre (1 hour) versus cities that did not have a CMN centre (2.8 hours). This time difference was not statistically significant.

### Treatment requested

**Figure 3:**
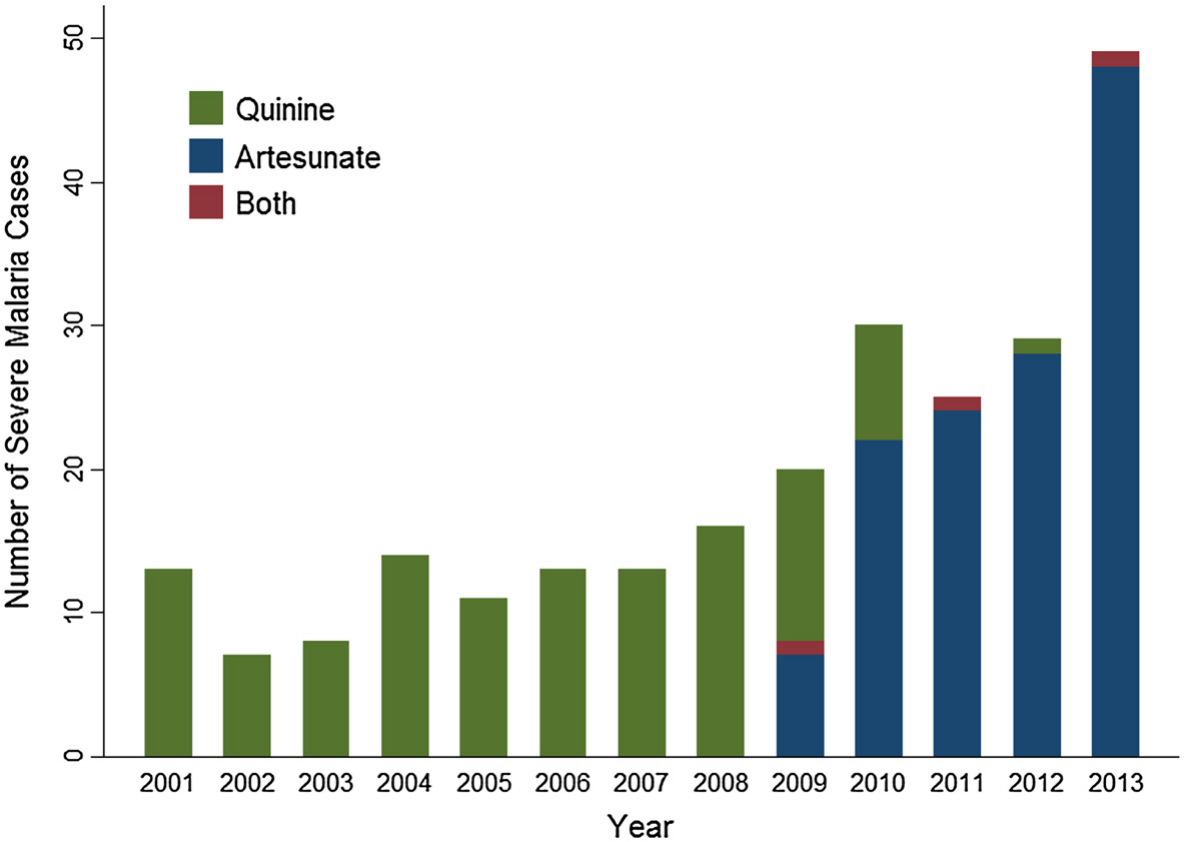
Severe malaria cases in Canada per year by type of IV parenteral therapy requested.

Among severe malaria cases, 116 (47%) were treated with IV quinine and 129 (52%) were treated with IV artesunate; both drugs were requested for three cases for which each was initially started on IV quinine, and were changed to IV artesunate therapy (1.0%). In one case treatment was switched to artesunate due to increased QTc with IV quinine, in two other cases quinine was given, since it was in stock, while awaiting transfer of artesunate from another hospital. Since the introduction of IV artesunate to Canada in 2009, the proportion of severe malaria cases treated with IV artesunate has increased rapidly as the treatment of choice (Figure 3).

### Symptom presentation

The most commonly reported clinical features of severe malaria include: hyperparasitaemia (70%), jaundice (29%), impaired consciousness or coma (27%) (Table 5). In addition, vomiting was reported as a symptom of malaria in 25% of severe malaria cases.

**Table 5:** **Severe malaria cases by indication for IV therapy**

**IV Indication**	**No./no.**	**(%)**
Hyperparasitemia	173/248	(70)
Jaundice	72/248	(29)
Impaired consciousness or coma	68/248	(27)
Vomiting	61/248	(25)
Circulatory collapse/shock	40/248	(16)
Hemoglobinuria	33/248	(14)
Acute kidney injury/renal failure	34/248	(13)
Metabolic acidosis/acidemia	33/248	(13)
Respiratory distress	29/248	(12)
Abnormal spontaneous bleeding/DIC	17/248	(7)
Severe anemia	17/248	(7)
Hypoglycemia	14/248	(6)
Respiratory failure/Pulmonary edema/ARDS	6/248	(2)
Multiple convulsions	4/248	(2)
Hyperlactataemia	4/248	(2)
Prostration	3/248	(1)
Other	41/248	(17)

### Hospitalization

CMN collects information on hospitalization, malaria complications and drug adverse events on Form B. Form B was completed for 43% of all patients accessing IV therapy, and in 60% of severe malaria cases (Table 6). There were no clinical or statistically significant differences between mBC and non-mBC cases for length of hospitalization, days to negative smear, length of intensive care unit (ICU) stay, administration of step-down therapy, supportive treatments, outcome or adverse events. Median time to a negative blood smear following treatment was three days among severe malaria patients. Cases spent a median of four days in hospital and a median one day in ICU. The majority of severe cases (87%) were prescribed at least one form of step-down therapy.

**Table 6:** **Outcomes of severe malaria cases** ^**a**^

	**All severe cases no./no. (%)**		**IV-Artesunate no./no. (%)**		**IV-Quinine no./no. (%)**		**P value**
Hospitalization, days, median (range) (n=133)	4	(0–28)	4	(0–28)	4	(0–27)	0.070
Intensive care unit stay, days, median (range) (n=134)	1	(0–19)	0	(0–17)	3	(0–19)	0.045
Negative blood smear, days, median (range) (n=104)	3	(0–21)	2	(0–8)	4	(1–21)	0.000
Stepdown therapy	130/150	(87)	88/99	(89)	42/51	(82)	0.265
IV adverse events	15/131	(11)	7/89	(8)	8/42	(19)	0.061
Minor complication	5/131	(4)	2/89	(2)	3/42	(7)	0.327
Serious complication	4/131	(3)	3/89	(3)	1/42	(2)	1.000
Other complication	6/131	(5)	2/89	(2)	4/42	(10)	0.083
Outcome							
Still hospitalized	27/143	(18)	16/92	(17)	11/51	(22)	0.514
Discharged	100/143	(70)	64/92	(70)	36/51	(71)	0.898
Deceased	3/143	(2)	1/92	(1)	2/51	(4)	0.257

^a^Three cases were started on IV quinine and transferred to IV artesunate. To simplify analysis, those cases were classified as having received IV artesunate therapy alone. None had adverse events or negative outcomes.

### Adverse events

Adverse events related to parenteral therapy were reported by 11% of severe malaria cases. Adverse events were classified as minor, serious or other (Table 6). Minor adverse events (fatigue, dizziness and occasional tinnitus) were reported in 7% of patients treated with quinine and 2% of those treated with artesunate. Serious adverse events were reported by 3% of cases (n=4) and involved a single case of hypotension and ischemic encephalopathy related to IV quinine, two cases of delayed haemolysis, and one case of early haemolysis associated with IV artesunate. Five per cent of severe cases reported adverse events classified as ‘other’ but were determined by the CMN physician (AM) to be unrelated to IV therapy alone and included nausea, vomiting and vivid dreams. No adverse events resulted in death. For a brief case summary of the severe drug-associated adverse events, see Additional file 2.

### Fatalities

Three fatalities occurred among severe and complicated malaria cases. All deaths occurred in mBC or VFR travellers. None of the reported deaths occurred in children. No fatalities were related to adverse events or complications from parenteral therapy. For a brief case summary of each fatality, see Additional file 2.

## Discussion

This study describes national trends, demographic characteristics, risk factors, and delays associated with all cases of severe and complicated malaria reported to CMN. CMN data demonstrate a gradual increase in annual incidence of severe malaria over time. This trend is consistent with results observed in the USA reported by the 2011 Morbidity and Mortality Weekly Report malaria surveillance summary [12]. The increase may be attributed to increased global travel and immigration [1,13].

In Canada there were slightly more severe malaria infections (18%) among children under age 18 years than in the USA (16%). Canada also had a higher percentage of severe cases with *P. falciparum* infection (94%), compared to 74% in the USA; however it is important to note that a species was identified and reported in 94% of Canadian cases versus only 77% in USA cases.

Most severe malaria cases identified Canada as their primary country of residence (80%) and in the USA, 75% identified the USA as their country of residence. Compared to the USA, a smaller proportion of Canadian travellers reported VFR as their reason for travel, 45% in Canada versus 59% in the USA.

The vast majority of severe and complicated malaria cases were reported from the most populated Canadian provinces of Quebec and Ontario, and occurred among travellers to and from Africa. These findings are consistent with the latest reports from Statistics Canada that state the highest proportions of new/recent immigrants to Canada reside in Quebec and Ontario, and originate from malaria-endemic regions such as Africa, Asia and Pacific, and South and Central Americas [14].

It was demonstrated that an individual’s country of birth is not a risk factor for malaria, indicating Canadian residents originating from mBC and non-mBC are at similar risk of malaria-related complications. Although more individuals from non-endemic birth countries reported accessing pre-travel advice, the overall use of appropriate and effective chemoprophylaxis was minimal at 13% (37/280) among all cases treated with IV therapy.

Experts believe diagnosis and treatment delays attributed to the non-specific symptomatology and unpredictable clinical complications of infection are the primary cause of severe infections in non-endemic regions, like Canada [15-18]. Patient delays of approximately three days occurred among reported cases. This finding highlights the importance of emphasizing malaria prevention and pre-travel health consultation prior to international travel, especially among VFR travellers heading to endemic regions [19]. Pre-travel consultations should inform travellers of malaria symptoms and outline the importance of seeking immediate medical attention should malaria symptoms develop. This same information should also be distributed to recent immigrants to Canada from malaria-endemic areas.

Even though the majority of severe malaria cases were diagnosed quickly and appropriately treated with parenteral therapy within a few hours of presentation to a Canadian physician, better health care provider knowledge of malaria symptoms (by travellers and health care practitioners) and reduced delays in access to effective therapy may reduce the number of cases that progress to severe disease. Furthermore, health care providers should educate Canadian VFR travelers of their malaria risk and loss of acquired malaria immunity over time during pre-travel medical visits.

Limitations of this analysis stem from missing data and gaps in the existing CMN dataset. A large number of case report forms were missing key information, and nearly half (43%) of the follow-up Form B were never submitted to CMN. As a result, presentation of a complete picture of malaria treatment procedures and outcomes, complications or adverse effects was not possible. Furthermore, given that Form B is collected 7 days following treatment initiation, cases of delayed hemolysis may not have been reported to CMN. Incomplete reporting of data highlights the importance of physician engagement in CMN surveillance activities in order to improve the availability and delivery of anti-malaria medications to patients. Additionally, the study sample was limited to patients treated with IV malaria therapies in Canada provided through the CMN. Therefore, the ability to capture information on additional cases of severe malaria with other treatments was limited, such as exchange therapy not possible.

## Conclusions

These findings demonstrate the utility of pre-positioning effective parenteral malaria therapy across the country through the CMN. The current trend of increased travel to endemic areas will increase the number of cases of severe malaria in Canada. These cases will continue to augment the need for access to parenteral therapy. There is a need for better travel health strategies to improve the uptake of pre-travel consultation and knowledge of malaria symptoms. These findings can inform the development of improved travel health practices and policies across Canada, and can be used to justify the development of such strategies in other non-endemic countries managing individuals with travellers’ malaria. Ideally, better education of travellers visiting malaria-endemic areas, especially VFR travelers returning to their country of origin, will result in faster treatment-seeking behaviour, leading to earlier diagnosis and effective therapy, thus minimizing the risk of severe disease.

## Additional files

Additional file 1:
**Forms used by physicians for the monitoring of parenteral therapy for severe malaria.**
Additional file 2:
**Adverse events and fatalities.**

## Abbreviations

CATMAT: Committee to Advise on Tropical Medicine and Travel
CDC: Centers for Disease Control and Prevention
CMN: Canadian Malaria Network
ICU: Intensive care unit
IV: Intravenous
mBC: Malaria-endemic birth country
MMWR: Morbidity and Mortality Weekly Report
non-mBC: Non-malaria endemic birth country
P.falciparum: Plasmodium falciparum
PHAC: Public Health Agency of Canada
VFR: Visiting friends and relatives
WHO: World Health Organization
