# Supplementary material for: Severe malaria in Canada, 2001–2013
Source: Malar J. 2015 Apr 11;14:151. doi: 10.1186/s12936-015-0638-y (PMC4418046; doi:10.1186/s12936-015-0638-y)
Supplement: Additional file 1: — Forms used by physicians for the monitoring of parenteral therapy for severe malaria. [file 12936_2015_638_MOESM1_ESM.doc]

# Additional file 1

# Form A

# Form B

# 
