# Supplementary material for: Severe malaria in Canada, 2001–2013
Source: Malar J. 2015 Apr 11;14:151. doi: 10.1186/s12936-015-0638-y (PMC4418046; doi:10.1186/s12936-015-0638-y)
Supplement: Additional file 2: — Adverse events and fatalities. [file 12936_2015_638_MOESM2_ESM.doc]

Additional file 2 – Adverse Events and Fatalities

# Adverse events

### Case 1: Hypotension and ischemic encephalopathy (2008)

In 2008, a male (71 years old) residing in Belgium, with a previous history of malaria, visited the Congo on business and subsequently travelled to Canada on 10 February. He did not use malaria prophylaxis. He became ill on 21 February and presented to a Quebec hospital on 23 February with impaired consciousness, renal injury, jaundice and acidosis. He was diagnosed with *Plasmodium falciparum* infection with 19% parasitaemia. Treatment was initiated on 25 February. He received a total 15 doses of IV quinine and was administered step-down therapy with doxycycline and oral quinine. A seven-day smear was negative. The patient was in the ICU for 19 days. Complications from malaria included convulsions, circulatory collapse, renal failure, anaemia, and multi-organ failure. The treating physician reported complications of IV quinine to be hypotension and ischemic encephalopathy.

### Case 2: Delayed haemolysis (2012)

On 16 December, 2012, a 44 year old Canadian-born male presented to a hospital in Alberta with *Plasmodium falciparum* hyperparasitaemia (2.7%), impaired consciousness, jaundice, DIC, acidosis and haemoglobinuria. He was working in the oil industry in Cameroon and was ill upon return to Canada on 13 December. He accessed pre-travel advice but his employer did not provide prophylaxis. His parasitaemia level climbed to 12.3% and he received his first dose of IV artesunate on 17 December. He was hospitalized for 28 days, with ten days in ICU. He received four doses of IV artesunate malaria therapy and three days of atovaquone/proguanil (Malarone) step-down therapy. He developed haemolysis on 21 December resulting in prolonged hospitalization. He was discharged from hospital on14 January, 2013.

### Case 3: Delayed hemolysis (2012)

A 44-year-old Canadian-born male presented to an Ontario hospital on 3 October, 2012 with vomiting, hyperparasitaemia (21.8%) and haemoglobinuria. He was in Sudan for ten days visiting friends and relatives, returning 21 September. He received pre-travel advice and used insect precautions but did not use prophylaxis. He became ill on 29 September, and a friend gave him atovaquone/proguanil four tabs plus doxycycline 100 mg plus artesunate/lumafantrine (Co-Artem) x one dose all at once. He was admitted to a local hospital on 3 October when he was found to have parasitaemia of 21.8%. He was treated with IV artesunate and then step-down therapy with 14 days of doxycycline and three days of Malarone. He developed delayed haemolysis which prolonged his stay in hospital and required multiple blood transfusions. He was discharged from hospital on 19 October, 2012 and had full recovery.

### Case 4: Early haemolysis (2013)

A 51-year-old Canadian-born male travelled to Burkina Faso for 13 days on business and returned to Canada ill on 21 July, 2013. He used Malarone chemoprophylaxis, but was not adherent. He presented to a Quebec hospital on 5 August, 2013 with respiratory distress, renal impairment, jaundice, acidosis, anaemia, haemoglobinuria, hyperlactaemia, thrombocytopaenia and hypoalbuminaenia. Laboratory tests confirmed *Plasmodium falciparum* with 10.25% parasitaemia that climbed to 15%. He was treated with three doses of IV quinine, which was stopped on 6 August when he was administered four doses of IV artesunate, nine days of clindamycin and ten days of doxycycline. Haemolysis from artesunate was reported. His smear was negative at day 3. He received transfusion with four units of blood. He was discharged from hospital on 23 August, 2013, and recovered from his illness.

# Fatalities

### Case 1 (2005)

In 2005, a 23-year old male visiting Canada from Haiti presented to a Quebec hospital with spontaneous bleeding, renal failure, pulmonary oedema, acadaemia, circulatory collapse, and jaundice. The patient became ill on 28 October, departed for Canada on 29 October, and presented to the hospital on 1 November. Blood smears identified a *Plasmodium* *falciparum* infection with 9.5% parasitaemia. IV quinine was readily available at the hospital, and was dispensed on 2 November (NB: IV artesunate was not available in Canada in 2005). On 3 November, the patient’s parasitaemia level decreased to 1%. The patient experienced impaired consciousness and multi-organ failure. On 4 November, following five doses of IV quinine and three days of concomitant clindamycin, the patient died. Death was attributed to malaria complications.

### Case 2 (2009)

A 52-year-old Canadian-born male travelled to Ghana to visit friends and relatives from 5 to 17 February, 2009. Although he obtained pre-departure travel advice, he did not take prophylaxis. He became ill on 1 March, and presented to a physician in Ontario on 4 March with impaired consciousness and acidaemia. Blood smears confirmed a mixed species infection with 1% parasitaemia for *Plasmodium* *falciparum*. He had no previous history of malaria. His first dose of IV quinine was administered on 5 March at the initial hospital and was then transferred to a tertiary care centre. The patient spent two days in ICU and received six doses of IV quinine (NB: IV artesunate was not available in Canada until mid-2009). He developed multi-organ failure and died on 8 March. Death was attributed to malaria complications.

### Case 3 (2013)

In 2013, a 56-year-old male, Kenyan-born Canadian resident travelled to Kenya to visit friends and relatives from 11 to 29 November. The patient presented to a hospital in Ontario on 4 December with impaired consciousness, respiratory distress and shock. Blood smears identified a *Plasmodium* *falciparum* infection with 1% parasitaemia. He was treated within 3.5 hours of presentation with IV artesunate. Four doses of IV-artesunate and three days of Malarone therapy were administered. The patient was thought to have a concurrent bacterial infection and also received Meropenem. He remained in the ICU on ventilation for 17 days. On 21 December, the patient died. The cause of death was attributed to sepsis and multi-organ failure secondary to a liver abscess.
